# Supplementary material for: Identification of Six Phytochemical Compounds from Asparagus officinalis L. Root Cultivars from New Zealand and China Using UAE-SPE-UPLC-MS/MS: Effects of Extracts on H2O2-Induced Oxidative Stress
Source: Nutrients. 2019 Jan 7;11(1):107. doi: 10.3390/nu11010107 (PMC6356627; doi:10.3390/nu11010107)
Supplement: Supplementary file 1 [file nutrients-11-00107-s001.pdf]

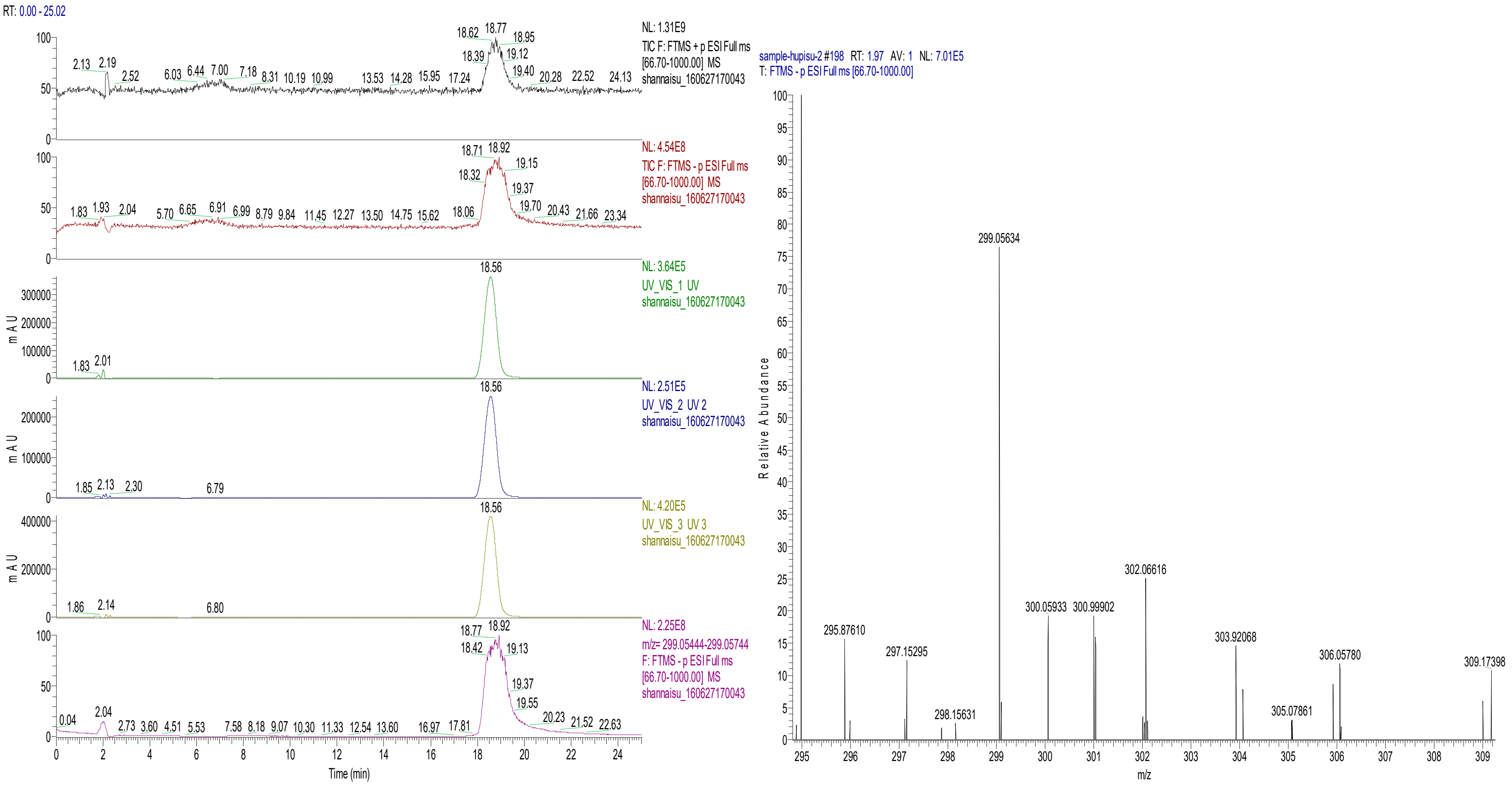

Figure 1. (a) Fragmentation patterns and full scan product ion mass spectra of $[M+H]^+$ ion of kaempferol standard.

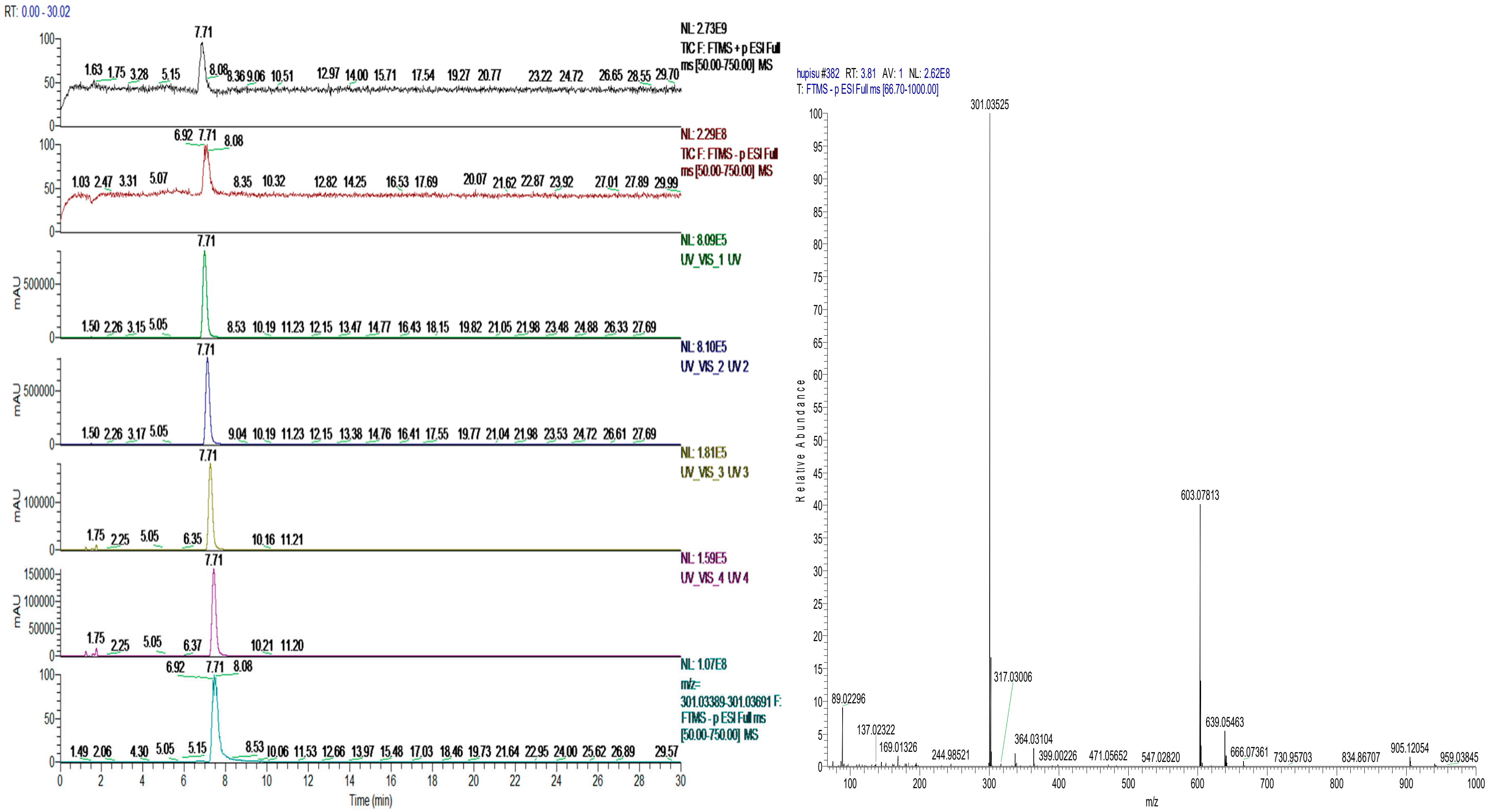

Figure S-1 (b) Fragmentation patterns and full scan product ion mass spectra of $[M+H]^+$ ion of quercetin standard.

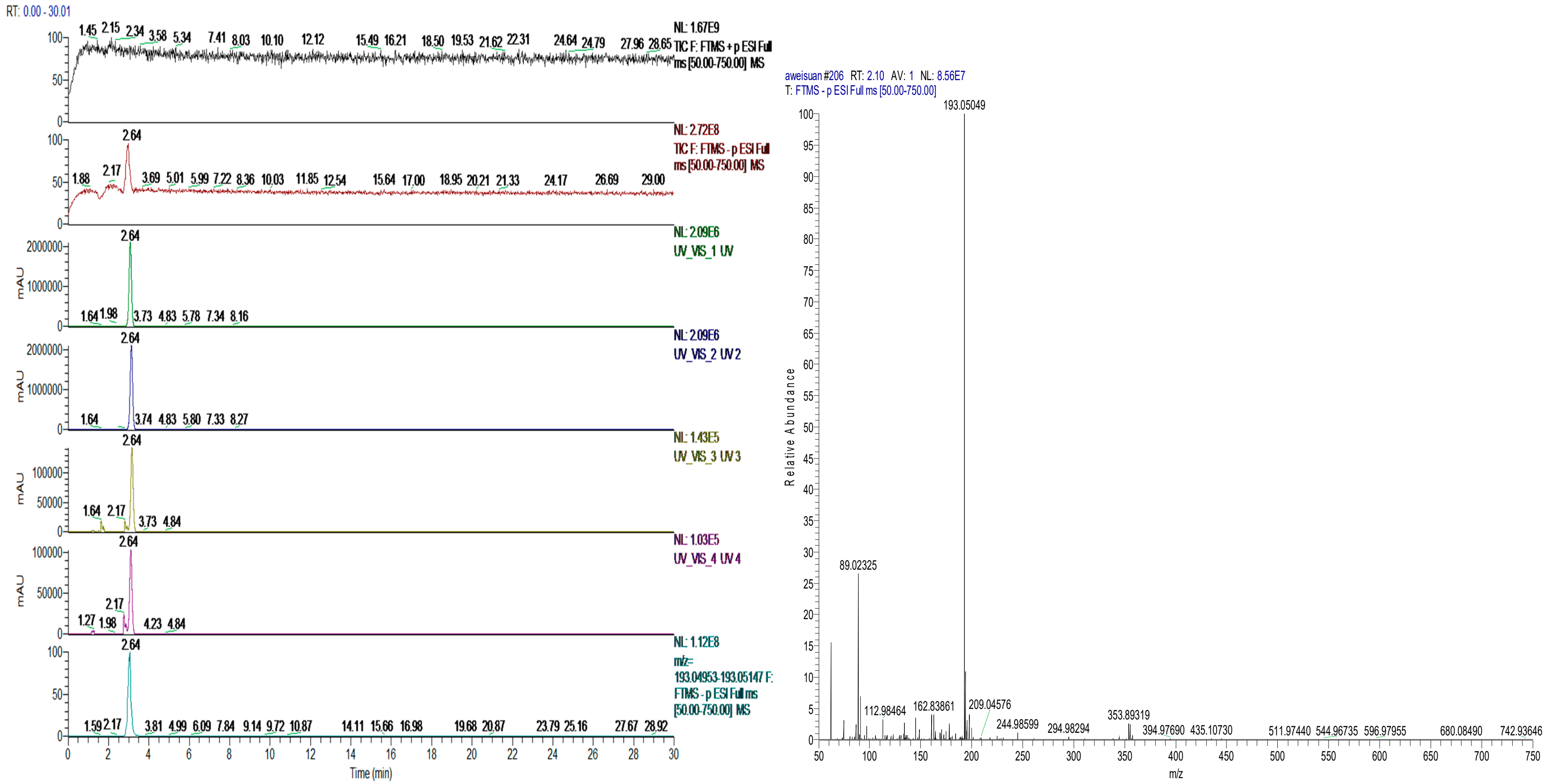

Figure S-1 (c) Fragmentation patterns and full scan product ion mass spectra of [M+H]$^{+}$ ion of ferulic acid standard.

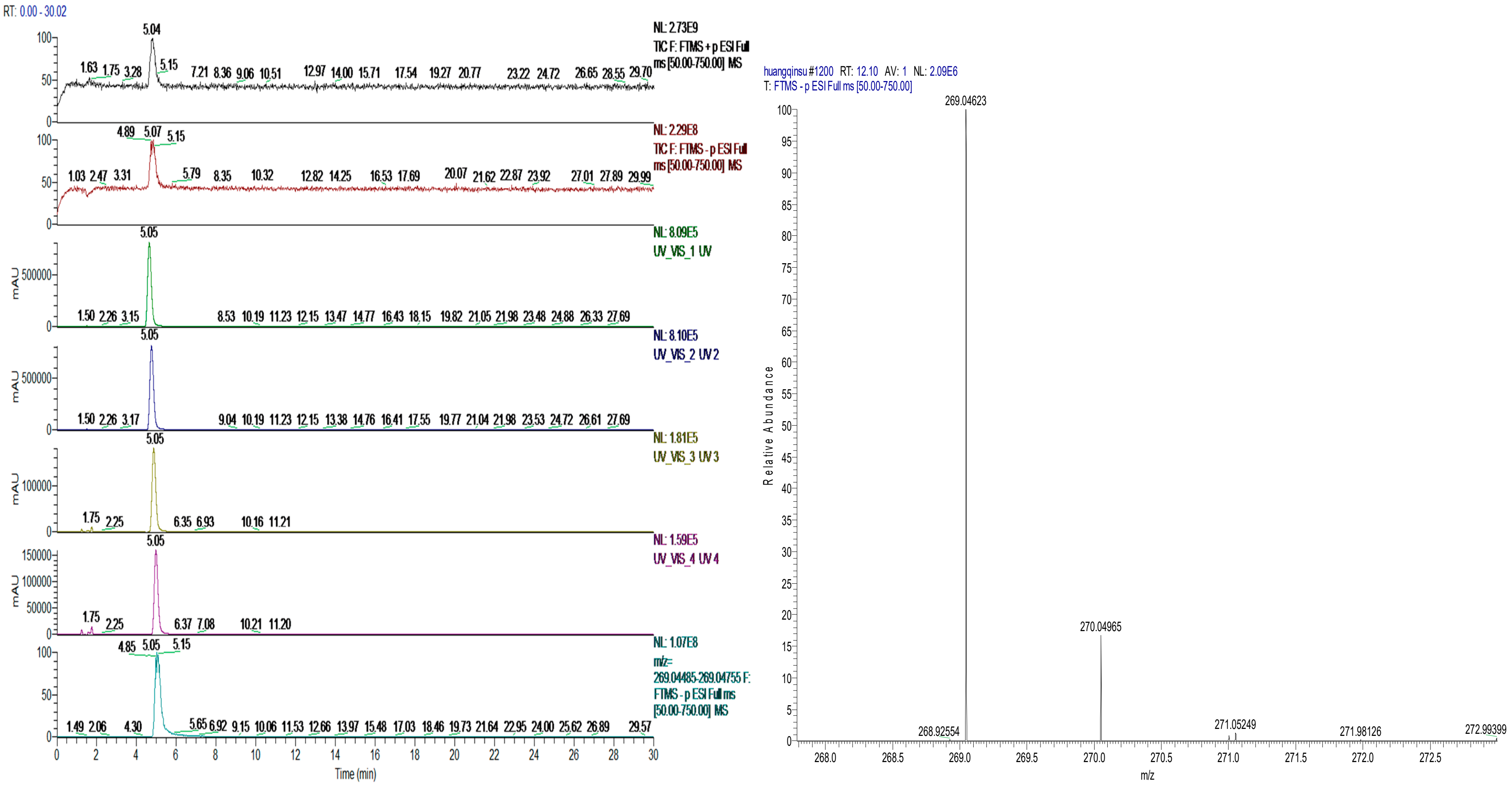

Figure S-1 (d) Fragmentation patterns and full scan product ion mass spectra of $[M+H]^+$ ion of apigenin standard.

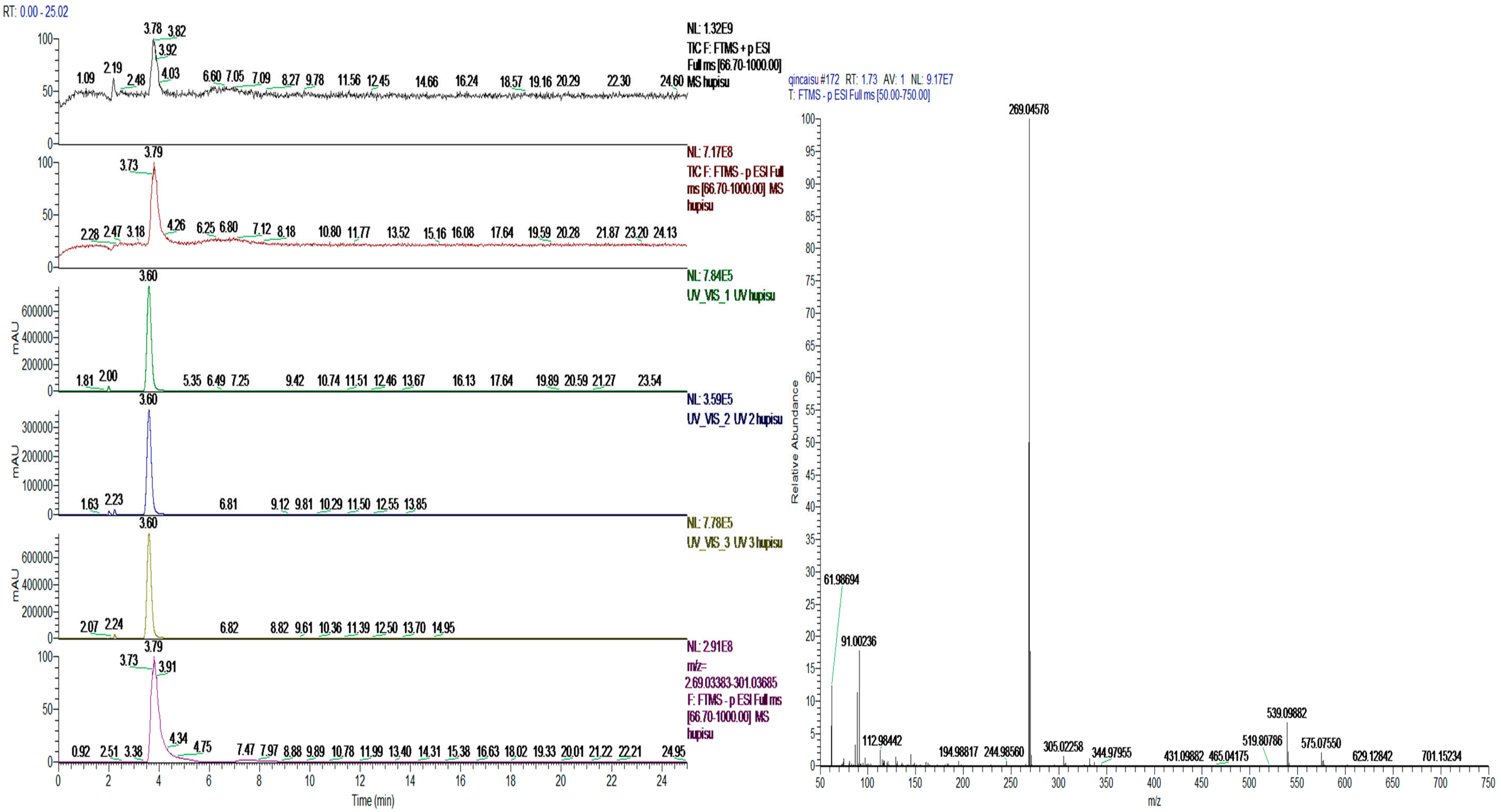

Figure S-1 (e) Fragmentation patterns and full scan product ion mass spectra of $[M+H]^+$ ion of baicalein standard.

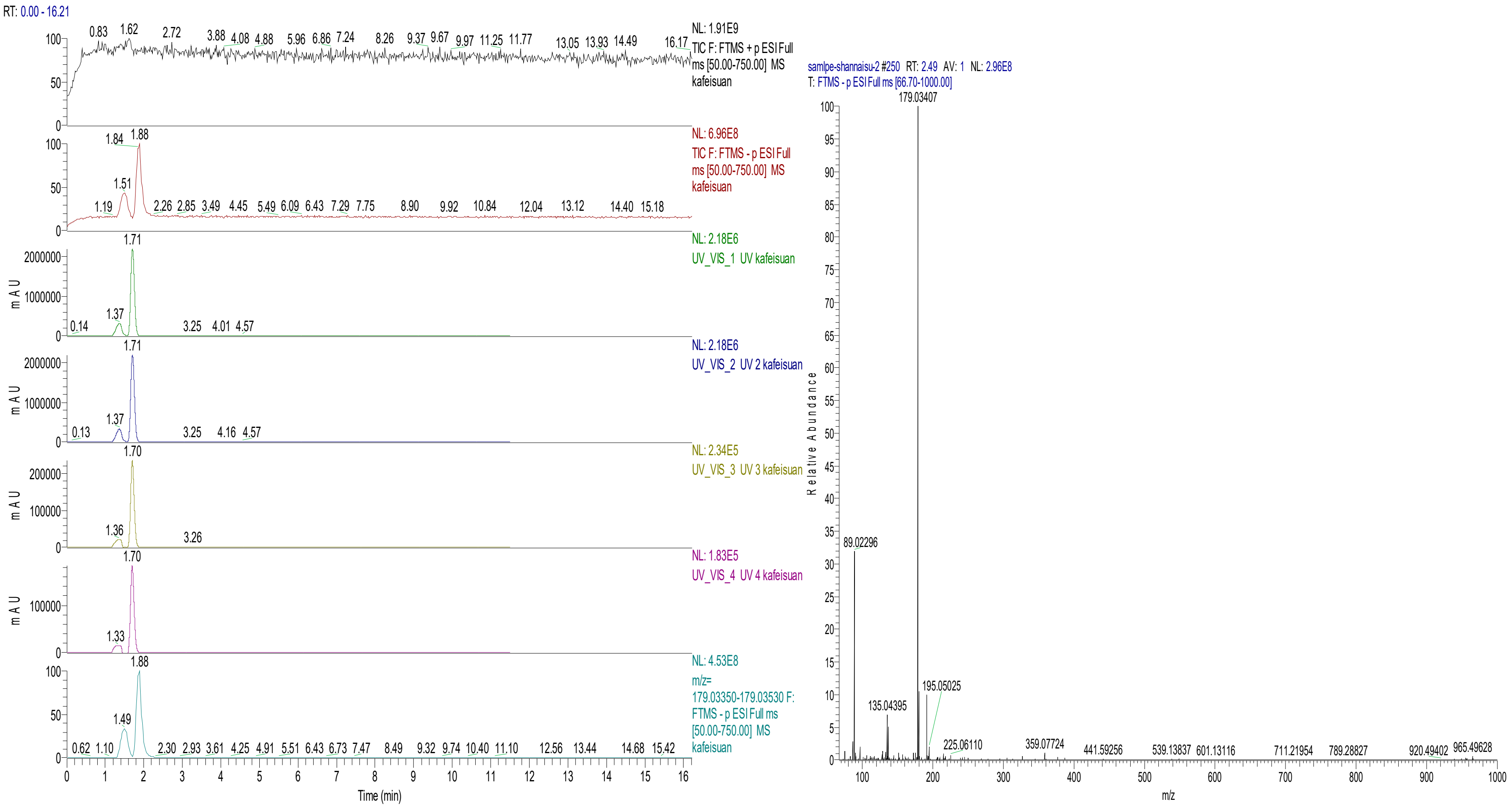

Figure S-1 (f) Fragmentation patterns and full scan product ion mass spectra of $[M+H]^+$ ion of caffeic acid standard.

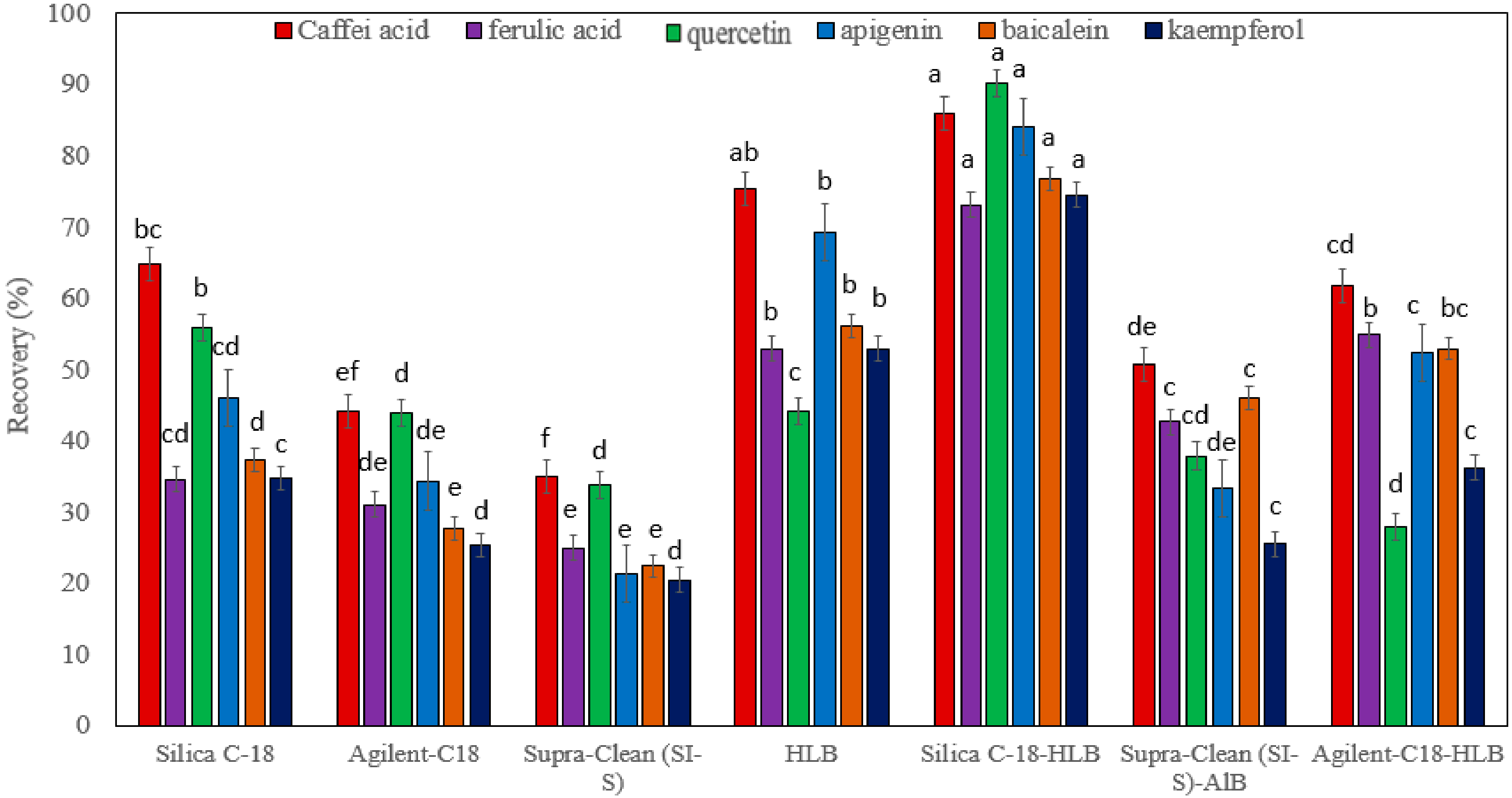

Figure S-2. Recovery of six compound in AR, calculated from the solvent-based standards, with different SPE cartridges using the optimized clean-up step. Data presented as mean ± standard error from 3 replicates (n=3). Bars with the different letters showed statistically significant difference (*P* < 0.05). The small letters mean that the recovery of same compounds compared with the different SPE columns.

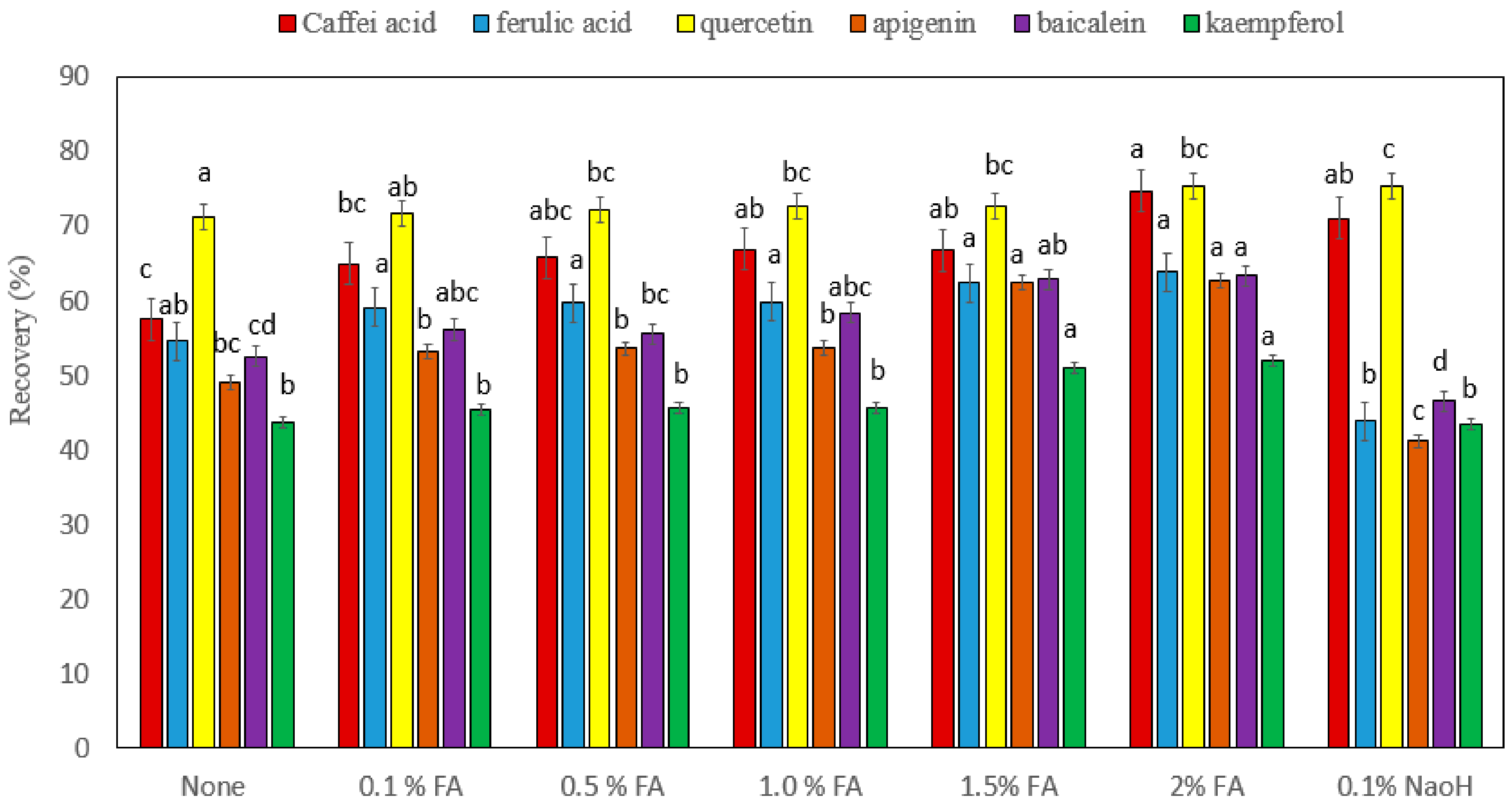

Figure S-3. Recovery of six compound in AR, calculated from the solvent-based standards, with the different concentration of formic acid (FA) extraction solution and salt-out step (0.1% NaOH). Data presented as mean ± standard error from three replicates (n=3) Bars with the different letters showed statistically significant difference (*P* < 0.05). The small letters mean that the recovery of same compounds compared with the different extraction conditions.

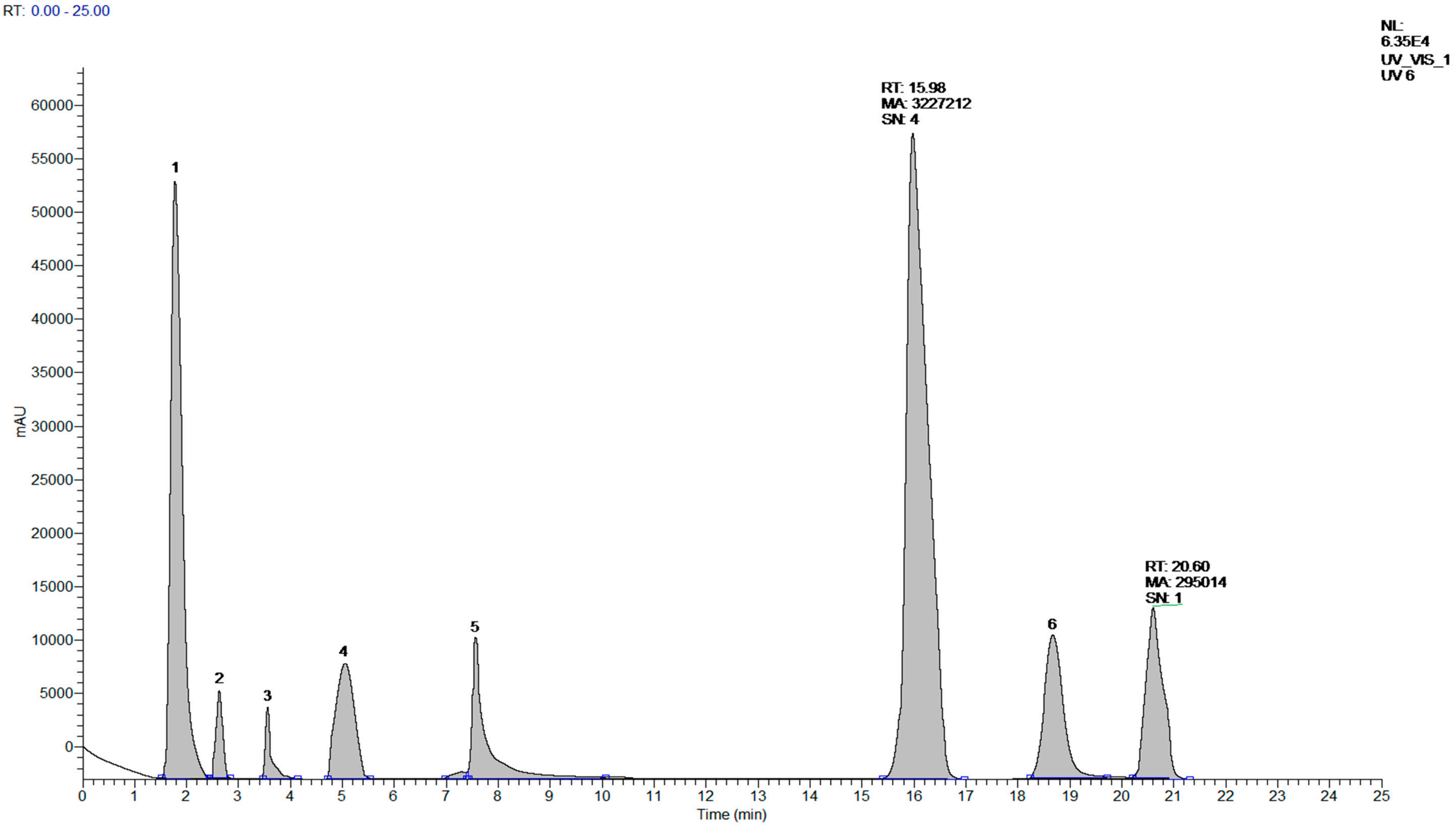

Figure S-4-G-C, UV and MS-SIM chromatograms of the six compounds in Chinese green AR. 1: Caffeic acid, 2: Quercetin, 3: Apigenin, 4: Ferulic acid, 5: Baicalein, 6: Kaempferol

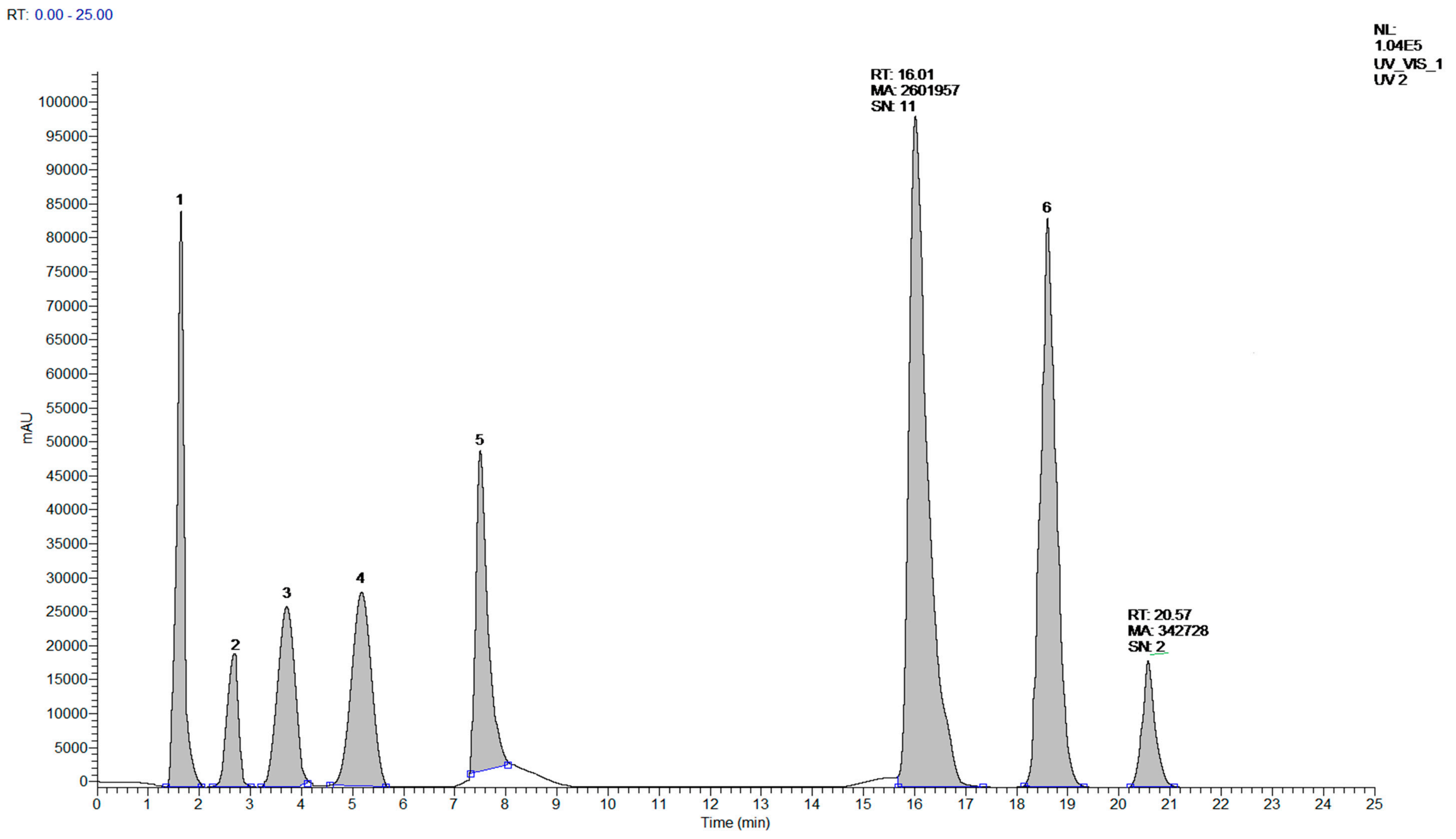

Figure S-4-G-N, UV and MS-SIM chromatograms of the six compounds in New Zealand AR. 1: Caffeic acid, 2: Quercetin, 3: Apigenin, 4: Ferulic acid, 5: Baicalein, 6: Kaempferol

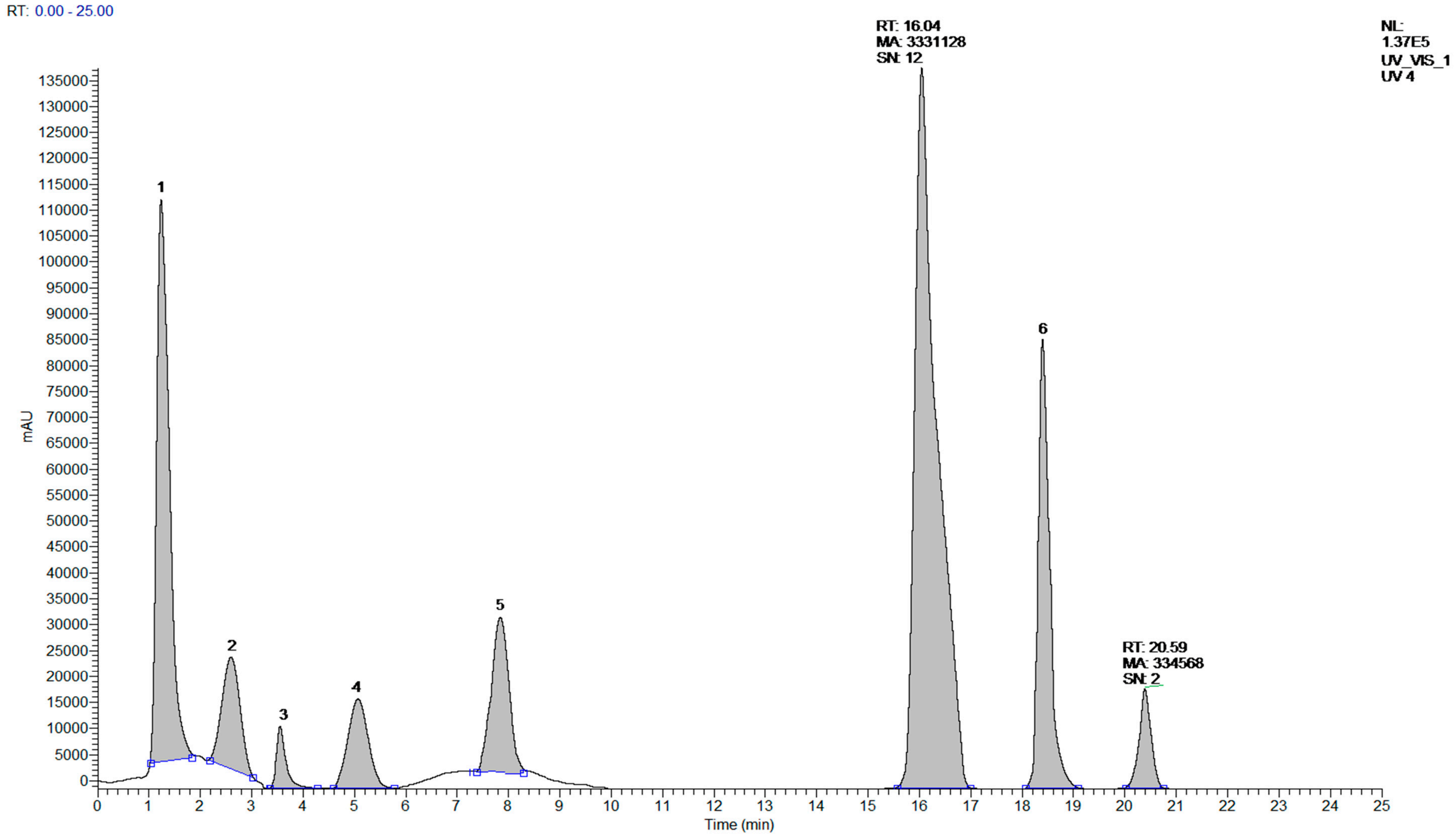

Figure S-4-P-C, UV and MS-SIM chromatograms of six compounds in Chinese purples AR. 1: Caffeic acid, 2: Quercetin, 3: Apigenin, 4: Ferulic acid, 5: Baicalein, 6: Kaempferol

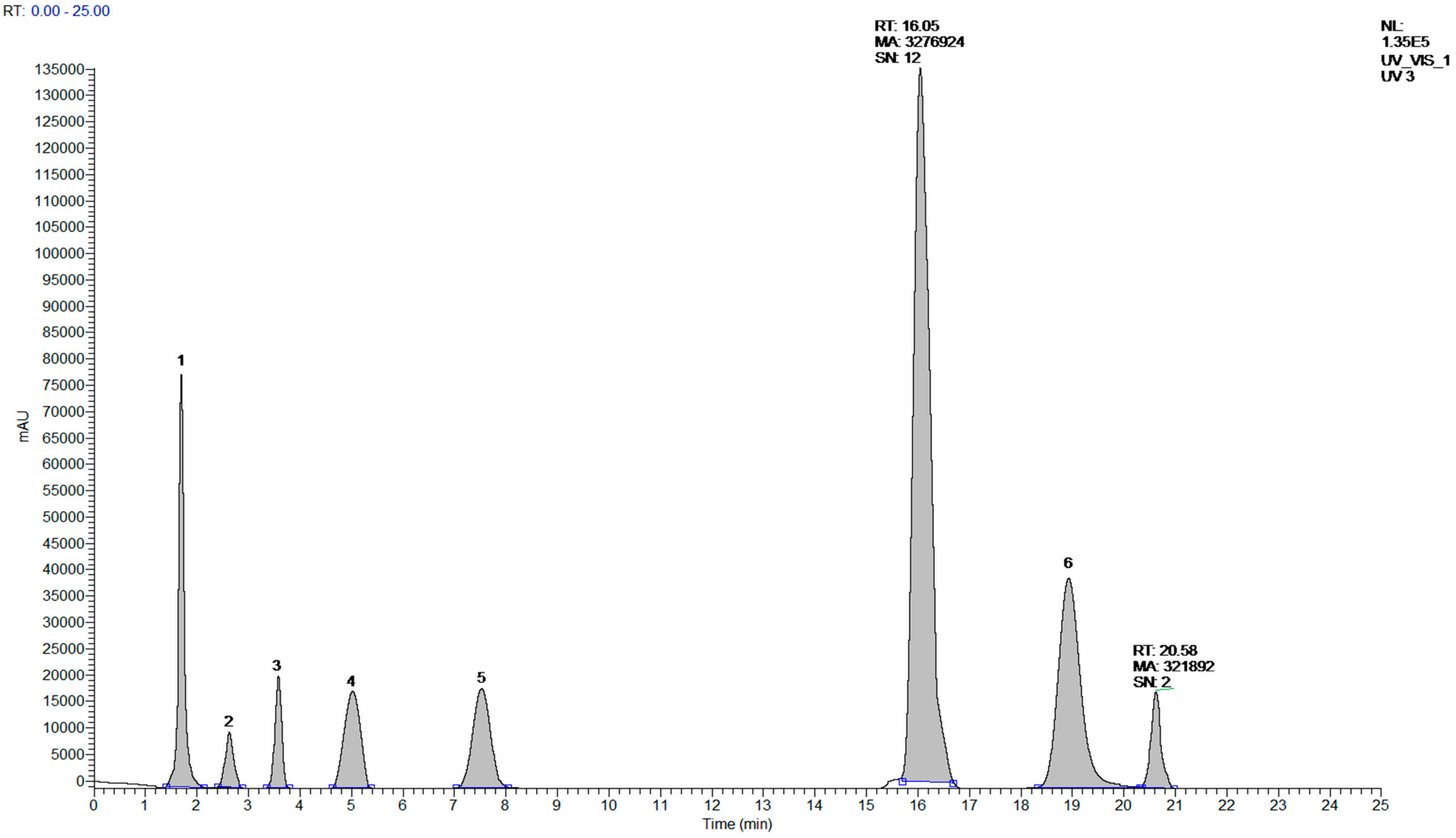

Figure S-4-P-NZ, UV and MS-SIM chromatograms of six compounds in New Zealand purple AR. 1: Caffeic acid, 2: Quercetin, 3: Apigenin, 4: Ferulic acid, 5: Baicalein, 6: Kaempferol
